# Gene-based association analysis of depression risk among European ancestry Parkinson’s disease patients

**DOI:** 10.1007/s00439-026-02877-8

**Published:** 2026-10-17

**Authors:** Hanwen Wang, Kimberly Paul, Cynthia Kusters, Christina M. Lill, Jin Zhou, Jeff Bronstein, Beate Ritz

**Affiliations:** 1 Department of Epidemiology, UCLA Fielding School of Public Health, Los Angeles, CA USA; 2 Department of Neurology, David Geffen School of Medicine, University of California, Los Angeles, CA USA; 3 Institute of Epidemiology and Social Medicine, University of Münster, Münster, Germany; 4 Ageing and Epidemiology Unit (AGE), School of Public Health, Imperial College London, London, UK; 5 Department of Biostatistics, UCLA Fielding School of Public Health, Los Angeles, CA USA; 6 Department of Environmental Health Science, UCLA Fielding School of Public Health, Los Angeles, CA USA

## Abstract

Depression is one of the most common and disabling non-motor symptoms in Parkinson’s disease (PD), yet its genetic basis remains poorly understood. Here, we investigate the genetic contributions to depression in PD by estimating the burden from common as well as low-frequency single nucleotide polymorphisms (SNPs) within genes previously implicated in PD or depression risk among European ancestry PD patients from three population-based studies. Using gene-based association analyses (SKAT, Burden, and SKAT-O) in the PASIDA, PEG, and PPMI studies, we evaluated SNP sets in gene regions previously linked to PD or depression. Among PD-related genes, meta-analysis identified a significant association between *KRTCAP2* and self-reported depression diagnosis in PD (SKAT-O *p* = 4.33 × 10⁻⁵), which remained significant after false discovery rate correction. A higher genetic burden in *KRTCAP2* was associated with 20% increased odds of depression (OR = 1.20, 95% CI: 1.04–1.38). *TRAF5* and *LRRK2* showed marginal associations. Among depression-related genes, *SPPL3* and *ERBB4* were associated with depression diagnosis history, and *FNIP2* was associated with current depressive symptoms. The PD polygenic risk score was not associated with depression in PD, while a higher depression polygenic risk score was significantly associated with increased odds of depression diagnosis in both cohorts and the meta-analysis (OR = 1.26, 95% CI: 1.13–1.40). Our findings highlight immune-related genes such as *KRTCAP2* and *TRAF5* as potential contributors to depression in PD. Additionally, the depression PRS shows statistically significant association with depression diagnosis in PD, suggesting that a general depression-related genetic liability contributes to this non-motor feature in PD. Together, these results suggest that depression in PD may involve both shared and disease-specific genetic mechanisms, offering new insights into its pathogenesis and potential therapeutic targets.

**Supplementary Information:**

The online version contains supplementary material available at https://doi.org/10.1007/s00439-026-02877-8.

## Introduction

Parkinson’s disease (PD) is the second most prevalent progressive neurodegenerative disorder, affecting an estimated 11.8 million individuals worldwide as of 2021, with the prevalence increasing by more than 270% since 1990 (Steinmetz et al. 2024). PD is characterized by the degeneration of dopaminergic neurons in the substantia nigra pars compacta (SNpc) (Fearnley and Lees 1991; Goiran et al. 2022), leading to motor symptoms such as bradykinesia, resting tremor, and rigidity (Moustafa et al. 2016). However, among non-motor symptoms, depression is one of the most disabling and highly prevalent features in all disease stages, including prodromally (Borgonovo et al. 2017; Ahmad et al. 2023). Depression also impacts the quality of life in PD considerably.

Despite numerous genome-wide association studies (GWAS) identifying common single nucleotide polymorphisms (SNPs) associated with PD (Do et al. 2011; Nalls et al. 2014, 2019) and with major depression in the general population (Howard et al. 2019; Ciuculete et al. 2020; Levey et al. 2021; Silveira et al. 2023), it remains unclear whether these genetic factors contribute to depression in PD. Common variants identified through GWAS often explain only a small proportion of trait heritability (Maher 2008; Manolio et al. 2009), leaving much of the genetic architecture of low-frequency and rare variants unaccounted for. Recent studies have suggested that incorporating a broader range of genetic variation in the analysis of complex traits, including low-frequency variants with minor allele frequencies (MAF) of 1% − 5% and potentially highly damaging rare variants (MAF < 1%), is necessary to better account for the unexplained heritability of complex traits (Auer and Lettre 2015; Bomba et al. 2017). A previous study used a GWAS and Mendelian Randomization approach to examine whether major depression and PD share common genetic variants (Reyes-Pérez et al. 2024), but did not focus on PD patients with depression or the possible role of low-frequency and rare variants, which are underexplored. Expanding analyses beyond common variants to include SNPs with lower allele frequency may provide a more complete picture of the genetic contributions to depression in PD.

Our study investigated the genetic basis of depression of PD among European ancestry PD patients from three cohorts of PD patients- the Parkinson in Denmark (PASIDA) study (Lee et al. 2016), the Parkinson’s, Environment, and Genes (PEG) study (Ritz et al. 2016), and the Parkinson’s Progression Markers Initiative (PPMI) study (Marek et al. 2011). These studies uniquely provide both PD diagnoses and detailed information on depression, as they collected depression diagnosis histories or current depressive symptoms. This allowed us to investigate the genetic basis of depression in these PD populations using several comprehensive tests (the Sequence Kernel Association Test (SKAT) (Wu et al. 2011), the Burden Test (Wu et al. 2011), and the Optimal Sequence Kernel Association Test (SKAT-O) (Lee et al. 2012). Using logistic regression models we estimated effect sizes for genes that were statistically significant according to these tests. Additionally, we generated polygenic risk scores (PRS) for PASIDA and PEG to assess whether the overall genetic burden for PD or depression was associated with a depression diagnosis history among PD patients.

Genetic factors associated with depression in PD may help us elucidate whether depression in PD shares genetic mechanisms with major depression in the general population or involves distinct PD-related pathways. This may also provide insights into the biological basis of mood disorders in PD and inform on more targeted interventions in this high-risk patient group.

## Methods

### Study population

This study is based primarily on European ancestry PD patients with depression information and necessary biological samples for genomic sequencing from three different studies: the Parkinson in Denmark (PASIDA) study, the Parkinson’s, Environment, and Genes (PEG) Study, and the Parkinson’s Progression Markers Initiative (PPMI) study. Additionally, given that PD patients of Hispanic ancestry represent the second most prevalent group in the PEG study, we included them for secondary analysis. Demographic characteristics and clinical features of each study cohort are summarized in Table 1.

#### Parkinson’s in Denmark (PASIDA) study

We identified European ancestry PD patients from the Danish National Hospital Register who had been discharged from one of ten major neurological centers with a primary PD diagnosis (International Classification of Diseases (ICD)-8 code 342 and ICD-10 code G20) between 1996 and 2009. Eligible patients were at least 35 years old and alive at the time of interview (2008–2010). Upper age cutoffs were also used to increase the likelihood of patient survival to follow-up. Further information on this patient population has been previously described (Kenborg et al. 2015; Lee et al. 2016). After further excluding subjects with incomplete information on the history of depression diagnosis and covariates of interest for our analyses here, there were 1,652 remaining PD patients in PASIDA, among whom 1,407 had genotyping data available and therefore could be included in the analyses. The final analytic sample comprised 1,407 European ancestry PD patients (60.1% male), with a mean age at PD diagnosis of 67.4 years (SD = 8.4) and a median PD duration at baseline of 6.0 years (IQR: 4.0–11.0).

The PASIDA study was approved by the Danish Data Protection Agency. Demographic characteristics for PD patients in PASIDA with and without genotyping data were largely comparable, with similar average ages at PD diagnosis of 67.4 (SD = 8.4) vs. 67.2 (SD = 9.4), although the proportion of males differed slightly, at 60.1% vs. 50.6%, respectively.

#### Parkinson’s, Environment, and Genes (PEG) study

From January 2001 to December 2007, in PEG 1, we identified new-onset, idiopathic PD patients diagnosed within 3 years of study enrollment through large medical groups, neurologists, and public service announcements in three agricultural counties in central California (Kern, Tulare, Fresno) (Costello et al. 2009; Nalls et al. 2014). Out of the 1,167 potential PD patients identified, 604 were excluded due to not meeting eligibility criteria: 397 did not have a PD diagnosis within 3 years before recruitment, 134 lived outside the three counties, 51 did not have PD, and 22 were too ill to participate. Among the remaining 563 potential cases, 90 were not examined by movement disorder specialists, with 56 declining exams or having moved away prior to being examined, and 34 who became too ill or died before their scheduled appointment. Of the 473 patients examined, 94 did not meet published criteria for idiopathic PD (Hughes et al. 1992), 13 were reclassified as not having idiopathic PD during follow-up, and 6 withdrew between examination and interview (Wang et al. 2011; Paul et al. 2019). Overall, the first wave of the PEG study consisted of 360 PD patients.

The second wave of new-onset, idiopathic PD patients (PEG2) was diagnosed within 5 years of study enrollment and recruited from 2011 to 2017 with the assistance of the California PD registry. Amongst the 734 eligible PD patients initially contacted, 601 PD patients were examined by neurologists at the University of California, Los Angeles (UCLA). After excluding subjects who were determined not to have PD and subjects with incomplete demographic information and medical history, the PEG 2 study consisted of 474 PD patients (Li et al. 2024).

Among the 834 eligible PD patients from the two waves of the PEG study, 762 had available genotyping data. Of these, 594 of European ancestry were included in the main analyses, with a median PD duration at baseline of 2.0 years (IQR: 1.0–4.0), while 142 of Hispanic ancestry were included in a secondary analysis only. The PEG study was conducted with the approval of the UCLA Institutional Review Board, and informed written consent was obtained from all participants. Demographic characteristics for European ancestry PD patients in PEG with and without genotyping data were similar, including an average age at PD diagnosis of 68.6 (SD = 10.1) vs. 67.9 (SD = 10.5), and 61.6% vs. 61.4% being male.

#### Parkinson’s Progression Markers Initiative (PPMI) study

PPMI was initiated in 2010 by the Michael J. Fox Foundation for Parkinson’s Research as a multi-site study of PD that is not population-based. The study initially enrolled 423 untreated PD participants aged 30 and older from 24 study sites located in the US (18 sites), in Europe (5 sites), and in Australia (1 site) (Marek et al. 2011). Detailed inclusion criteria of the PD cases in the PPMI study have been described previously (Marek et al. 2018). Of the 423 eligible PD patients, 383 European ancestry PD patients had genotyping data available for inclusion in the analyses, with a median PD duration at baseline of 0.4 years (IQR: 0.2–0.7). The demographic characteristics of the European PD patients in PPMI with and without genotyping data were similar, with an average age at PD diagnosis of 61.3 (SD = 9.5) vs. 62.0 (SD = 11.2), and 65.5% vs. 70.0% being male.

### Data collection and assessment

#### Assessment of depression

Self-reported physician diagnoses of clinical depression were obtained through interviews conducted by trained staff in the PASIDA and PEG studies. Additionally, the PEG and PPMI studies gathered data on current depressive symptoms using the 15-item version of the Geriatric Depression Scale (GDS) (Sheikh and Yesavage 1986), which measures self-reported depressive symptoms over the previous week, with the total score ranging from 0 to 15. A score of 5 or above is generally considered indicative of the presence of current clinical depression in PD patients (Weintraub et al. 2006). Notably, a previous study evaluating self-administered depression measures in the PEG study suggested that the GDS-15 is a superior screening tool for detecting depression in PD in clinical settings (Thompson et al. 2011).

#### Selection of genes of interest

We selected 171 gene regions based on the locations of SNPs previously associated with PD, as identified through the PDgene database and large GWAS studies (Satake et al. 2009; Simón-Sánchez et al. 2009; International Parkinson Disease Genomics Consortium [IPDGC] 2011; Do et al. 2011; Lill et al. 2012; Pankratz et al. 2012; Nalls et al. 2014, 2019). These genes were selected to investigate whether variants in PD-related gene regions contribute to depression risk in PD patients, given that PD pathology itself may play a role in the development of depression. Additionally, we selected 315 gene regions previously associated with depression in general (Wilkinson et al. 2011; Howard et al. 2019; Kim et al. 2020; Reyes-Pérez et al. 2024) to examine whether common genetic risk factors for depression in the general population also contribute to depression in PD. These gene regions encompass the full transcribed region of the genes, including exons, introns, and untranslated regions (UTRs).

#### Collection of biosamples and genotyping data

DNA from participants across the three studies was extracted from peripheral whole blood drawn at the baseline visit.

DNA samples from PEG and PASIDA studies were genotyped with the Illumina Infinium Global Screening Array. Raw data processing, including clustering and genotype calling, was performed in GenomeStudio v2.0.4 (Illumina, Inc.) using the “GSAsharedCUSTOM_20018389_A6” manifest, which provided annotations for a total of 696,375 variants. GenTrain v3.0 (Illumina, Inc.) was used for automatic clustering and genotype calling. Variants with GenTrain scores < 0.7 (*n* = 110,579), samples with a call rate < 95%, and those with a GenCall score < 0.7 were excluded at this stage.

Further quality control (QC) was performed using PLINK v1.9. We excluded SNPs with a missing genotype rate > 2%, SNPs failing Hardy-Weinberg equilibrium (HWE) tests (*P* < 5e-6), and variants with MAF < 1%. This QC process yielded 495,338 SNPs across 1,866 PEG and 3,486 PASIDA samples suitable for imputation. Haplotype phasing was first performed with ShapeIT v2.r790 (Delaneau and Marchini 2014), referencing the HRC.r1.2016 panel (McCarthy et al. 2016). Imputation was then conducted via the Michigan Imputation Server, using Minimac3 (Das et al. 2016) with GRCh37.p13 as the reference genome. Post-imputation, we retained autosomal SNPs with a Minimac3 r^2^ ≥0.3. Genotyped variants were hard-called using an 80% imputation probability threshold, resulting in 3,891,048 bi-allelic variants. Further filtering removed variants with MAF < 1%, HWE *P* <1e-7, and call rates < 90%, leaving 7,306,154 variants in PASIDA and 7,370,482 variants in PEG after imputation.

For PPMI, all available biobanked DNA specimens were genotyped using two arrays: Illumina Infinium ImmunoChip and NeuroX. Genotyping followed Illumina’s standard protocol, with initial QC assessing individual genotype success rates (≥ 95%) and consistency between reported and genotyped sex (Nalls et al. 2016).

Before statistical analyses, additional QC was performed on the variant call format (VCF) files using PLINK v2.0. We excluded variants lacking genotype (GT) fields and those with multi-character allele codes. To focus on variants within gene regions of interest, we filtered the genotyping data to retain only SNPs located within the 171 PD-related and 315 depression-related gene regions, as defined by previous large-scale GWAS. Variants with missing call rates < 95% were retained.

For the PASIDA and PEG studies, SNP locations were determined using GRCh37.p13 as the reference genome, while for the PPMI study, SNP locations were mapped to GRCh38.p14. In order to conduct the meta-analyses of gene associations with dichotomized GDS scores representing current depressive symptoms among PD patients from the PEG and PPMI studies, we used Lift Genome Annotations by UCSC Genome Browser to align SNP data across different human genome reference versions (Shumate and Salzberg 2021).

We further performed linkage disequilibrium (LD) pruning using a 50-kilobase window, a step size of 5, and an r² threshold of 0.8. This LD pruning was conducted separately for PASIDA and PEG PD patients and for PEG and PPMI PD patients, ensuring retention of consistent SNPs across datasets with the same measurement of depression phenotypes used in statistical analyses. For PD-related gene regions, 38,537 SNPs across 168 genes remained in the PASIDA and PEG datasets for analyses based on clinical depression diagnosis history, while 55,358 SNPs across 166 genes remained in the PEG and PPMI datasets for analyses based on the dichotomized GDS. For depression-related gene regions, 188,636 SNPs across 314 genes were retained in the PASIDA and PEG datasets for the depression diagnosis phenotype, while 270,449 SNPs across 307 genes were retained in the PEG and PPMI datasets for the dichotomized GDS.

Finally, we performed an A-transpose transformation to convert the genotype matrix from variant-major to sample-major format, generating data compatible with downstream statistical analyses in R.

#### Collection of demographics

Demographic information for PD patients in the PEG and PPMI studies was collected through structured phone interviews conducted by trained interviewers. For the PASIDA study, demographic information was obtained from the records of the Danish National Hospital Register. Information on genetic ancestry was inferred using the Structure program v2.3.4 (Pritchard et al. 2000), grouping subjects into five superpopulations: Africans (AFR), Admixed Americans (AMR), East Asians (EAS), Europeans (EUR), and South Asians (SAS). For the purposes of this study, we retained only the European ancestry PD patients in the main analyses to maintain a more homogeneous study sample.

#### Calculation of the weighted PRS

To investigate the cumulative genetic risk associated with depression in PD, we calculated PRS for each European ancestry PD patient in the PASIDA and PEG studies based on the genome-wide genotype data. PRS analyses were restricted to PASIDA and PEG as these were the only study cohorts with available depression diagnosis history data; PPMI participants were assessed for depressive symptoms only using the GDS, as a PRS generated for clinically diagnosed depressionwould allow for phenotypic mismatch when applied to current depressive symptoms.

Separate PRS were computed for PD genetic risk and major depression genetic risk using published scores from the PGS Catalog. The PD PRS was calculated using PGS000903 (Nalls et al. 2019), and the depression PRS was calculated using PGS004759 (Truong et al. 2024). Both scores were applied to PASIDA and PEG genotype data and z-standardized prior to analysis.

### Statistical analyses

We employed several comprehensive tests (Sequence Kernel Association Test (SKAT) (Wu et al. 2011), Burden Test (Wu et al. 2011), Optimal Sequence Kernel Association Test (SKAT-O) (Lee et al. 2012) to investigate the associations between SNPs grouped by gene regions and depression phenotypes. Given the abundance of rare variants across the genome, these statistical approaches allow us to overcome challenges due to low statistical power and high multiple testing penalties of simpler regression models. Specifically, we used the “SKATBinary_Robust” function in the SKAT R package, as it is more scalable and accurate for binary traits. By modifying the function’s options, we performed the SKAT test (method = “SKAT”) with the ρ parameter for the compound symmetric correlation structure kernel set to 0, the Burden test (method = “Burden”) with ρ = 1, and the optimal SKAT test (method = “SKATO”) where the minimum p-value was computed and selected from eight values of ρ = (0, 0.1^2^, 0.2^2^, 0.3^2^, 0.4^2^, 0.5^2^, 0.5, 1) to provide the best type I error control (Lee et al. 2012). We applied the default weight scheme (weights.beta = c(1,25) to increase the weight of rare variants while giving non-zero weights for variants with MAF 1% − 5%. Additionally, we used the default weighted linear kernel (kernel = “linear.weighted”) and the default imputation method (impute.method = “bestguess”), which imputes missing genotypes as most likely values (0, 1, or 2) (Wu et al. 2011).

After performing the analyses separately in each of the three study cohorts, we conducted meta-analyses. To investigate the association between genes and self-reported diagnoses of clinical depression, we performed meta-analyses with PD patients from PASIDA and PEG. For dichotomized GDS scores representing current depressive symptoms, we performed meta-analyses with PD patients from PEG and PPMI. These analyses were conducted using the “MetaSKAT_wZ” function in the MetaSKAT R package (Lee et al. 2013). By adjusting the options in the “MetaSKAT_wZ” function, we performed the meta-SKAT test (method = “davies”, r.corr = 0), the meta-Burden test (method = “davies”, r.corr = 1), and the meta optimal SKAT test “meta-SKATO” (method = “optimal”). To account for heterogeneous genetic effects of SNP sets across studies, we set the argument “is.separate = TRUE”. We adjusted for age and sex in all analyses.

We also performed sensitivity analysis on the SNPs in PD-related genes in PD patients from PEG and PASIDA by stratifying by sex, age (≥ 60, < 60), and including only PD patients with a self-reported depression diagnosis occurring prior to the PD diagnosis. We adjusted for age and sex in all strata, except sex-specific strata analyses were adjusted only for age. We also examined associations between top genes from the main results and self-reported depression diagnosis history among 142 Hispanic-ancestry PD patients to assess whether associations observed in the European ancestry group replicated.

After adjusting the p-values for multiple testing using the Benjamini-Hochberg procedure (Benjamini and Hochberg 1995) to control the false discovery rate (FDR), we identified one gene that consistently showed statistically significant results. For this gene, we performed logistic regression to obtain an effect size represented by an odds ratio (OR), which involved generating a burden score using the SNPs included within this gene region, while adjusting for the covariates age and sex.

To assess associations of the PD PRS and depression PRS with a history of clinical depression diagnosis in PD, we used separate logistic regression models for each PRS to estimate ORs with 95% CIs while adjusting for age and sex. Meta-analysis across PASIDA and PEG was conducted using the metagen function from the meta R package, applying the random effects model with restricted maximum-likelihood estimation to account for between-cohort heterogeneity.

### Comparability across study cohorts

To ensure analytical comparability, meta-analyses were conducted only for cohorts sharing the same depression assessment. PASIDA and PEG, paired for analyses of a depression diagnosis history, were well-matched in the genotyping platform, and demographic characteristics, including similar mean age at PD diagnosis (67.4 vs. 68.6 years) and sex distribution (60.1% vs. 61.6% male), but they differed in disease duration at baseline (median 6.0 vs. 2.0 years). PEG and PPMI, paired for current depressive symptoms measured by GDS, showed greater differences in study design, genotyping platform, age at PD diagnosis (68.6 vs. 61.3 years), and disease duration at baseline (median 2.0 vs. 0.4 years). Heterogeneity in genetic effects across cohorts was accounted for analytically by setting the argument “is.separate = TRUE” in all MetaSKAT analyses, and differences in reference genome builds were addressed through SNP liftover prior to meta-analysis.

## Results

The European ancestry PD patients in this study were predominantly 65 years of age or older, with approximately 60% being male in the PASIDA, PEG, and PPMI studies (Table 1). In the PEG study, one PD patient was missing a GDS score and was excluded from analyses involving GDS.

Table 2 presents the top eight genes identified from the primary analyses examining the association between SNPs in PD-related gene regions and self-reported depression diagnosis history among European ancestry PD patients. The table is sorted by SKAT-O p-values from the meta-analysis, with results showing marginal significance (*p* < 0.05). For comparison, p-values from the SKAT and Burden tests in both the PASIDA and PEG cohorts are also provided, though not all reached the same level of significance. *KRTCAP2* emerged as the most consistent finding with statistically significant associations in the meta-analysis (SKAT-O: *p* = 4.33E-05, SKAT: *p* = 2.78E-05) and both PASIDA (SKAT-O: *p* = 0.001, SKAT: *p* = 3.61E-04) and PEG (SKAT-O: *p* = 0.001, SKAT: *p* = 0.004); it remained statistically significant after FDR adjustment. *TRAF5* also showed significant associations in the meta-analysis (SKAT-O: *p* = 0.002, SKAT: *p* = 0.001), though this association was predominantly driven by the PASIDA PD patients (SKAT-O: *p* = 0.004, SKAT: *p* = 0.002). Similarly, *LRRK2* showed a marginally statistically significant association in the meta-analysis (SKAT-O: *p* = 0.009, SKAT: *p* = 0.005) and PASIDA (SKAT: *p* = 0.011), but only a suggestive association in PEG (SKAT: *p* = 0.077). Apart from these three genes, *VCAM1*, *FGF20*, *FAM171A2*, *TIRAP*, and *BST1* showed nominal statistical significance (*p* < 0.05) in at least one test, though associations were less consistent across individual studies. Complete results for all PD-related genes we assessed, including marginal and FDR-adjusted p-values in PASIDA, PEG, and meta-analytic results, are available in Supplementary Table 1.

**Table 1:** Demographics of European ancestry PD patients from the PASIDA, PEG, and PPMI studies (*n* = 2,384)

	Total	With depression diagnosis history	No depression diagnosis history	Higher depressive symptoms (GDS ≥ 5)	Lower depressive symptoms (GDS < 5)
**Parkinson in Denmark (PASIDA) study**	(*n* = 1,407)	(*n* = 277)	(*n* = 1,130)	-	-
*Sex*					
Male	845 (60.1)	152 (54.9)	693 (61.3)	-	-
Female	562 (39.9)	125 (45.1)	437 (38.7)	-	-
*Age at PD diagnosis (years)*	67.4 (8.4)	67.3 (7.7)	67.5 (8.6)	-	-
*PD duration at baseline (years) (median [IQR])]*	6.0 [4.0, 11.0]	6.0 [3.0, 11.5]	7.0 [4.0, 11.0]	-	-
*Age at depression diagnosis (years)*	-	55.9 (13.1)	-	-	-
**Parkinson’s, Environment, and Genes (PEG) study**	(*n* = 594)	(*n* = 185)	(*n* = 409)	(*n* = 181)	(*n* = 412)
*Sex*					
Male	366 (61.6)	106 (57.3)	260 (63.6)	116 (64.1)	249 (60.4)
Female	228 (38.4)	79 (42.7)	149 (36.4)	65 (35.9)	163 (39.6)
*Age at PD diagnosis (years)*	68.6 (10.1)	66.1 (10.0)	69.8 (10.0)	68.1 (11.0)	68.8 (9.7)
*PD duration at baseline (years) (median [IQR])]*	2.0 [1.0, 4.0]	2.0 [1.0, 4.0]	2.0 [1.0, 3.0]	2.0 [1.0, 4.0]	2.0 [1.0, 3.0]
*Age at depression diagnosis (years)*	-	52.0 (18.8)	-	-	-
**Parkinson’s Progression Markers Initiative (PPMI) study**	(*n* = 383)	-	-	(*n* = 49)	(*n* = 334)
*Sex*					
Male	251 (65.5)	-	-	27 (55.1)	224 (67.1)
Female	132 (34.5)	-	-	22 (44.9)	110 (32.9)
*Age at PD diagnosis (years)*	61.3 (9.5)	-	-	60.7 (9.9)	61.4 (9.4)
*PD duration at baseline (years) (median [IQR])]*	0.4 [0.2, 0.7]	-	-	0.3 [0.2, 0.6]	0.4 [0.2, 0.7]

Sex reported as n (%); Age reported as mean (SD)

**Table 2:** Marginal P-values from SKAT-O, SKAT, and Burden test for SNPs in PD-related gene regions associated with self-reported depression diagnosis history among European ancestry PD patients from the PASIDA (*n* = 1,407) and PEG (*n* = 594) studies

Genes	Meta analysis			PASIDA			PEG		
	SKAT-O	SKAT	Burden	SKAT-O	SKAT	Burden	SKAT-O	SKAT	Burden
KRTCAP2	4.33E-05^‡^	2.78E-05^‡^	0.002	0.001***	3.61E-04****	0.176	0.001**	0.004	0.001***
TRAF5	0.002**	0.001****	0.722	0.004	0.002**	0.426	0.205	0.116	0.625
LRRK2	0.009	0.005	0.471	0.018	0.011	0.331	0.136	0.077	0.904
VCAM1	0.011	0.344	0.006	0.005	0.242	0.003	0.708	0.632	0.502
FGF20	0.021	0.126	0.011	0.022	0.057	0.014	0.547	0.848	0.351
FAM171A2	0.027	0.125	0.014	0.230	0.131	0.227	0.027	0.294	0.014
TIRAP	0.029	0.014	0.632	0.020	0.010	0.784	0.614	0.399	0.660
BST1	0.034	0.856	0.018	0.381	0.876	0.229	0.039	0.531	0.022

FDR adjusted p-values: < 0.01 ‘‡’; < 0.10 ‘****’; 0.15 ‘***’; < 0.20 ‘**’

Total *n* = 2,001

The results from the sensitivity analyses with SKAT-O p-values from the meta-analysis of PASIDA and PEG for subgroups defined by sex, age, and depression diagnosis timing are shown in Table 3. KRTCAP2 remained statistically significantly associated with a history of depression diagnosis among male PD patients (*n* = 1,211; SKAT-O: *p* = 0.001, SKAT: *p* = 0.001), PD patients age ≥ 60 years (*n* = 1,653; SKAT-O: *p* = 2.05E-04, SKAT: *p* = 1.04E-04), and those with depression diagnosed prior to PD only (*n* = 1,862; SKAT-O: *p* = 0.009, SKAT: *p* = 0.005). Associations were no longer statistically significant in smaller subgroups of female PD patients (*n* = 790) or those aged < 60 years (*n* = 348). Similarly, *TRAF5* showed statistically significant associations among male PD patients (SKAT-O: *p* = 0.033, SKAT: *p* = 0.017), those age ≥ 60 years (SKAT-O: *p* = 0.008, SKAT: *p* = 0.004), and those with depression prior to PD only (SKAT-O: *p* = 0.013, SKAT: *p* = 0.007) but not for female PD patients and younger PD patients. *LRRK2* showed a marginally significant association in PD patients aged ≥ 60 years (SKAT-O: *p* = 0.011, SKAT: *p* = 0.006), but no association in other subgroups. Full details on sensitivity analyses, including marginal and adjusted SKAT-O, SKAT, and Burden test results within each subgroup in the meta-analysis of PASIDA and PEG, are provided in Supplementary Table 2.

The odds ratios (ORs) from the logistic regression analysis assessing the association between burden scores of SNPs within *KRTCAP2* and self-reported depression diagnosis history among European ancestry PD patients in the PASIDA and PEG studies are presented in Table 4.

**Fig. 1:**
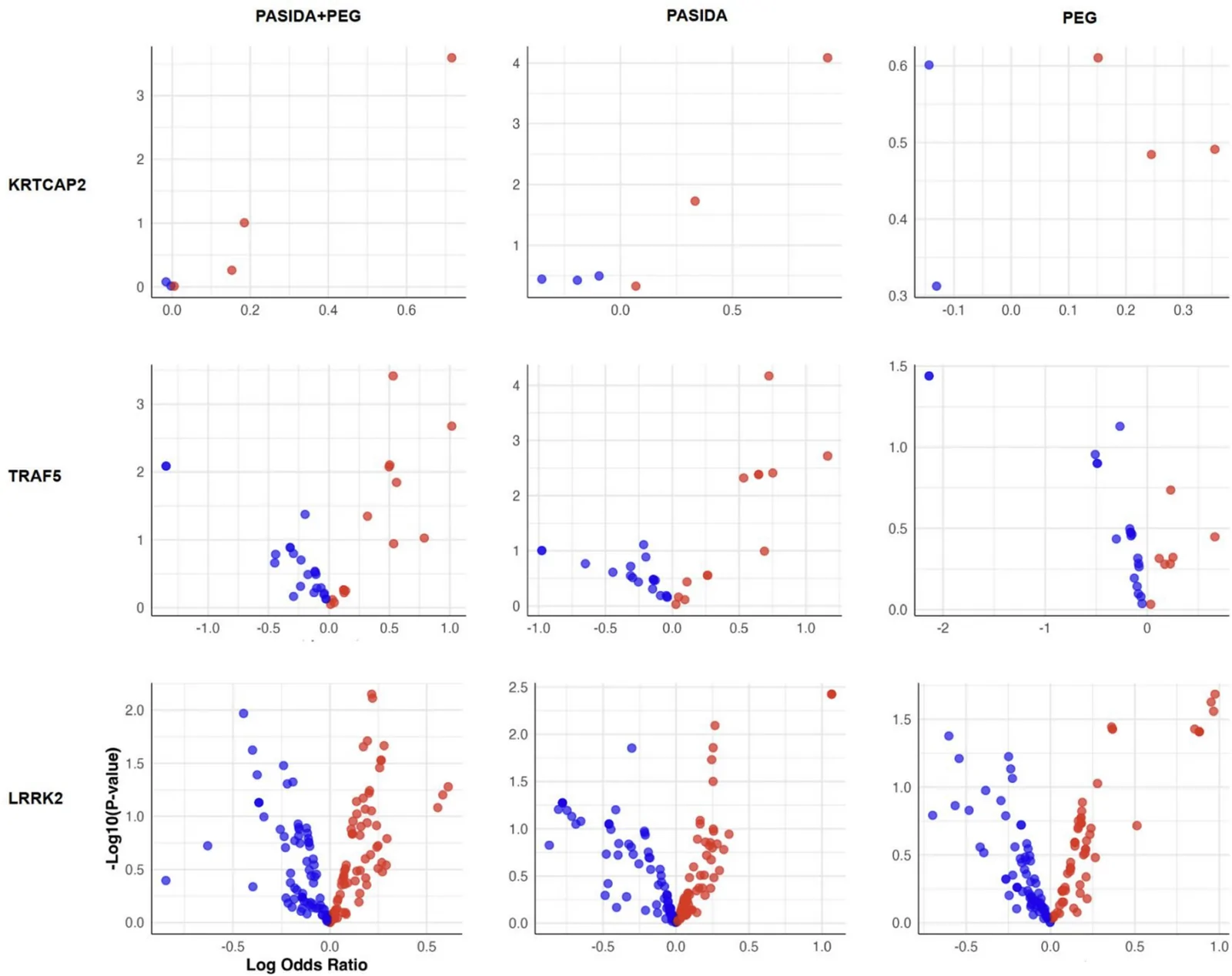
SNP effects in *KRTCAP2*, *TRAF5*, and *LRRK2* in PASIDA, PEG, and the combined meta-analysis (PASIDA + PEG). Red and blue points indicate SNPs with positive and negative log odds ratios, respectively. The bidirectional distribution of SNP effects in *TRAF5* and *LRRK2* illustrates the heterogeneity in effect directions within these gene regions

Overall, European ancestry PD patients with a higher burden score had an approximately 20% higher odds of having been diagnosed with depression compared to those with a lower burden score (meta-analysis OR = 1.20, 95% CI: 1.04–1.38). The association size was similar in male PD patients (1.25 [1.04–1.50]) and older PD patients (1.18 [1.01–1.39]) but did not reach statistical significance in female PD patients (1.12 [0.89–1.42]), PD patients younger than 60 (1.27 [0.90–1.79]), or those with depression diagnosed prior to PD only (1.10 [0.92–1.31]). We did not calculate ORs for *TRAF5* and *LRRK2*, as SNPs within these gene regions exhibited opposing effect estimate directions, making it challenging to derive a meaningful summary for effect size . As illustrated in Fig. 1, the volcano plots for *TRAF5* and *LRRK2* in PASIDA, PEG, and the combined meta-analysis (PASIDA + PEG) revealed that different SNPs within each gene region were associated with increased or decreased odds of depression. This bidirectional pattern suggests heterogeneity in genetic effects, further supported by the non-significant p-values observed in the Burden tests. Given the lack of a consistent directional effect, calculating a single OR for these gene regions would not reflect the gene’s relationship with depression in PD.

Among Hispanic ancestry PD patients in the PEG study (*n* = 142), none of the top eight genes identified in the European ancestry analysis showed statistically significant associations with a depression diagnosis history (Supplementary Table 3). While *VCAM1* showed a marginally statistically significant p-value in the SKAT (*p* = 0.024), SKAT-O (*p* = 0.033), and Burden test (*p* = 0.040) (OR = 1.15 [0.96–1.40]) among Hispanic ancestry PD patients, none of the genes demonstrated statistically significant associations with self-reported depression diagnosis history after FDR adjustment. The lack of replication may reflect limited statistical power due to the smaller sample size in the Hispanic ancestry group, genetic heterogeneity, or population-specific differences in genetic architecture.

In the meta-analysis of the associations between SNP sets grouped by PD-related gene regions and current depressive symptoms, represented by the GDS score, five genes—*BIRC3*, *LY96*,* SPTSSB*,* TNFSF13B*, and *LAT*— showed marginally statistically significant p-values in the SKAT, SKAT-O, or Burden tests, although none remain statistically significant after FDR adjustment (Table 5). Complete results for all analyzed PD-related genes and the GDS score, including marginal and FDR-adjusted p-values in PEG, PPMI, and the meta-analysis are available in Supplementary Table 4.

Lastly, we used the same analytical approach to focus on the depression-related gene regions. While no genes remained statistically significant after FDR adjustment, several genes showed consistent associations across different tests and cohorts. In the analyses of associations between SNPs in depression-related gene regions and depression diagnosis history, *SPPL3* had consistently lower p-values in the meta-analysis (SKAT-O: 0.005, Burden: 0.003) and in individual studies (PASIDA: SKAT-O: 0.073, Burden: 0.052; PEG: SKAT-O: 0.031, Burden: 0.016). Similarly, *ERBB4* showed marginally consistent results in the meta-analysis (SKAT-O: 0.007, Burden: 0.003) and individual studies (PASIDA: SKAT-O: 0.035, Burden: 0.018; PEG: SKAT-O: 0.088, Burden: 0.080) (Table 6). *FNIP2* emerged as the most consistently identified gene for current depressive symptoms, with SKAT-O p-values of 0.012 in the meta-analysis, 0.070 in PEG, and 0.040 in PPMI (Table 7). Results for all depression-related genes we evaluated in relation to depression diagnosis history and GDS score, including marginal and FDR-adjusted p-values across PASIDA, PEG, PPMI, and the meta-analyses are available in Supplementary Tables 5 and Supplementary Table 6.

**Table 3:** Marginal P-values from the meta analyses SKAT-O, SKAT, and Burden tests for SNPs in PD-related gene regions associated with self-reported depression diagnosis history among subgroups of European ancestry PD patients from the PASIDA (*n* = 1,407) and PEG (*n* = 594) Studies

Genes	Males (PASIDA: 845; PEG: 366)			Females (PASIDA: 562; PEG: 228)			≥ 60 years old (PASIDA: 1,171; PEG: 482)			< 60 years old (PASIDA: 236; PEG: 112)			Depression Diagnosis Prior to PD Diagnosis (PASIDA: 1,306; PEG: 556)		
	SKAT-O	SKAT	Burden	SKAT-O	SKAT	Burden	SKAT-O	SKAT	Burden	SKAT-O	SKAT	Burden	SKAT-O	SKAT	Burden
KRTCAP2	0.001****	0.001****	0.003*	0.145	0.083	0.173	2.05E-04†	1.04E-04†	0.009	0.050	0.192	0.026	0.009	0.005	0.048
TRAF5	0.033	0.017	0.659	0.113	0.062	0.936	0.008	0.004	0.488	0.695	0.465	0.782	0.013	0.007	0.946
LRRK2	0.109	0.060	0.398	0.184	0.103	0.945	0.011	0.006	0.528	0.743	0.750	0.511	0.095	0.052	0.746
VCAM1	0.120	0.699	0.065	0.065	0.461	0.035	0.010	0.137	0.005	0.497	0.877	0.312	0.186	0.668	0.104
FGF20	0.129	0.284	0.070	0.172	0.363	0.095	0.007	0.153	0.003	0.262	0.149	0.792	0.068	0.225	0.036
FAM171A2	0.005	0.098	0.002	0.918	0.735	0.929	0.002**	0.011	0.002	0.542	0.342	0.425	0.023	0.064	0.015
TIRAP	0.547	0.407	0.341	0.043	0.021	0.624	0.215	0.117	0.655	0.825	0.603	0.705	0.015	0.007	0.492
BST1	0.180	0.959	0.100	0.181	0.693	0.102	0.096	0.904	0.053	0.400	0.429	0.238	0.021	0.491	0.011

FDR adjusted p-values: < 0.05 ‘†’; < 0.10 ‘****’; 0.15 ‘***’; < 0.20 ‘**’; < 0.25 ‘*’

(Total *n* = 2,001)

**Table 4:** Odds ratios (ORs) and 95% confidence intervals (CIs) for the association between Burden scores of KRTCAP2 SNPs and self-reported depression diagnosis history among European ancestry PD patients from the PASIDA (*n* = 1,407) and PEG (*n* = 594) studies (Total *n* = 2,001)

	Meta analysis	PASIDA	PEG
Total¹	1.20 (1.04, 1.38)	1.19 (0.99, 1.42)	1.21 (0.95, 1.53)
Male²	1.25 (1.04, 1.50)	1.27 (1.00, 1.59)	1.22 (0.89, 1.66)
Female²	1.12 (0.89, 1.42)	1.09 (0.81, 1.45)	1.19 (0.81, 1.75)
Age ≥ 60¹	1.18 (1.01, 1.39)	1.18 (0.96, 1.44)	1.19 (0.91, 1.55)
Age < 60¹	1.27 (0.90, 1.79)	1.25 (0.81, 1.88)	1.30 (0.72, 2.38)
Depression prior to PD ¹	1.10 (0.92, 1.31)	1.04 (0.82, 1.31)	1.17 (0.90, 1.51)

^1^Adjusted for age and sex

^2^Adjusted for age

Logistic regression models revealed no significant association between the PD PRS (PGS000903) and a history of clinical depression diagnosis in PD in either cohort or the meta-analysis (Meta: OR = 0.94 [0.82, 1.08], PASIDA: OR = 1.00 [0.87, 1.14], PEG: OR = 0.87 [0.72, 1.04]). In contrast, a higher depression PRS (PGS004759) was significantly associated with increased odds of depression diagnosis in both PASIDA (OR = 1.25 [1.10, 1.43], *p* = 0.0009) and PEG (OR = 1.27 [1.06, 1.52], *p* = 0.0094), with a consistent meta-analytic estimate (OR = 1.26 [1.13, 1.40], *p* < 0.0001).

**Table 5:** Marginal P-values from SKAT-O, SKAT, and Burden test for SNPs in PD-related gene regions associated with current depressive symptoms represented by GDS score among European ancestry PD patients from the PEG (*n* = 593) and PPMI (*n* = 383) studies (Total *n* = 976)

Genes	Meta analysis			PEG			PPMI		
	SKAT-O	SKAT	Burden	SKAT-O	SKAT	Burden	SKAT-O	SKAT	Burden
BIRC3	0.009	0.007	0.044	0.008	0.004	0.075	0.568	0.707	0.355
LY96	0.016	0.570	0.008	0.144	0.488	0.081	0.034	0.712	0.018
SPTSSB	0.027	0.014	0.446	0.027	0.014	0.888	0.179	0.359	0.100
TNFSF13B	0.039	0.045	0.045	0.175	0.175	0.113	0.033	0.017	0.239
LAT	0.041	0.802	0.021	0.133	0.613	0.074	0.230	0.963	0.136

**Table 6:** Marginal P-values from SKAT-O, SKAT, and Burden test for SNPs in depression-related genes associated with self-reported depression diagnosis History among European ancestry PD patients from the PASIDA (*n* = 1,407) and PEG (*n* = 594) studies (Total *n* = 2,001)

Genes	Meta analysis			PASIDA			PEG		
	SKAT-O	SKAT	Burden	SKAT-O	SKAT	Burden	SKAT-O	SKAT	Burden
HLA-B	0.003	0.025	0.002	0.001	0.014	0.001*	0.743	0.554	0.521
SPPL3	0.005	0.107	0.003	0.073	0.083	0.052	0.031	0.449	0.016
CARM1P1	0.006	0.053	0.004	0.158	0.230	0.092	0.013	0.026	0.011
ERBB4	0.007	0.285	0.003	0.035	0.575	0.018	0.088	0.086	0.080
HLA-DQA1	0.010	0.067	0.005	0.010	0.025	0.006	0.445	0.760	0.286
LINC01876	0.019	0.047	0.019	0.016	0.015	0.031	0.496	0.806	0.298
HSPE1-MOB4	0.021	0.010	0.155	0.051	0.025	0.437	0.146	0.083	0.174
FAM120AOS	0.026	0.452	0.013	0.052	0.426	0.026	0.393	0.458	0.236
TOPAZ1	0.029	0.014	0.369	0.143	0.102	0.130	0.026	0.013	0.606
FNIP2	0.029	0.069	0.024	0.004	0.023	0.003	0.915	0.873	0.728
KCNG2	0.035	0.018	0.657	0.046	0.024	0.590	0.209	0.183	0.139
MOB4	0.035	0.017	0.279	0.107	0.055	0.491	0.092	0.047	0.368
SLC44A4	0.038	0.308	0.019	0.050	0.178	0.027	0.530	0.731	0.340
ATP1A3	0.041	0.407	0.020	0.027	0.479	0.013	0.496	0.303	0.608
BMERB1	0.048	0.890	0.023	0.111	0.912	0.057	0.369	0.537	0.211
CCS	0.048	0.024	0.165	0.010	0.005	0.066	1.000	0.957	0.827
FDR adjusted p-values: < 0.25 '*'									

**Table 7:** Marginal P-values from SKAT-O, SKAT, and Burden test for SNPs in depression-related gene regions associated with current depressive symptoms represented by GDS score among European ancestry PD patients from the PEG (*n* = 593) and PPMI (*n* = 383) studies (Total *n* = 976)

Genes	Meta Analysis			PEG			PPMI		
	SKAT-O	SKAT	Burden	SKAT-O	SKAT	Burden	SKAT-O	SKAT	Burden
SPPL3	0.004	0.382	0.373	0.702	0.486	0.494	0.358	0.212	0.539
OR12D3	0.007	0.704	0.285	0.358	0.705	0.220	0.705	0.493	0.929
HIVEP2	0.009	0.299	0.164	0.606	0.421	0.388	0.300	0.216	0.201
FNIP2	0.012	0.024	0.013	0.070	0.038	0.155	0.040	0.174	0.021
DRD2	0.026	0.116	0.015	0.064	0.141	0.038	0.346	0.321	0.203
RN7SKP120	0.028	0.263	0.160	0.205	0.205	0.205	0.539	0.539	0.539
KIF15	0.029	0.359	0.653	0.313	0.183	0.983	0.499	0.965	0.305
STK19	0.029	0.956	0.305	1.000	0.930	0.780	0.238	0.775	0.135
TAOK3	0.030	0.408	0.948	0.424	0.461	0.269	0.183	0.354	0.109
C3orf84	0.031	0.799	0.015	0.112	0.540	0.059	0.222	0.986	0.122
OTX2	0.035	0.232	0.695	0.541	0.430	0.346	0.121	0.065	0.505
SCYL1	0.043	0.919	0.533	0.771	0.941	0.552	0.680	0.460	0.833
LST1	0.044	0.730	0.407	0.640	0.880	0.436	0.289	0.170	0.762
COP1	0.049	0.485	0.029	0.153	0.469	0.098	0.249	0.536	0.162
SLC30A9	0.049	0.037	0.591	0.095	0.052	0.972	0.340	0.201	0.356

## Discussion

We evaluated the genetic contributions of SNPs in genes related to PD or depression in general to self-reported clinical depression diagnoses and current depressive symptoms, as measured by GDS. Our results identified several interesting gene candidates, including *KRTCAP2*,* TRAF5*,* LRRK2*,* SPPL3*,* ERBB4*, and *FNIP2* as being associated with depression phenotypes among European ancestry PD patients in multiple studies.

### Key findings and biological implications

*KRTCAP2* emerged as the most consistent and statistically significant finding for its associations with self-reported depression diagnosis in the meta-analysis and individual studies, retaining its statistical significance after FDR adjustment. *KRTCAP2* encodes keratinocyte-associated protein 2, a non-catalytic subunit of the oligosaccharyltransferase (OST) complex. This complex catalyzes the N-glycosylation of proteins, a critical process for protein folding and cell-cell communication within the endoplasmic reticulum (ER) lumen (Shibatani et al. 2005; Wilson et al. 2011). N-glycosylation is highly responsive to inflammatory changes and plays a central role in immune function. From innate to adaptive immunity, this process may amplify inflammation (Radovani and Gudelj 2022), which is increasingly recognized as a contributing factor to mood disorders, including depression, particularly in the context of neurodegenerative diseases such as PD (Paul et al. 2022; Foley et al. 2023). Our findings suggest that *KRTCAP2* may influence depression through immune-related pathways. Further investigation into its precise role in neuroinflammation and mood regulation in PD is needed.

*LRRK2*, encoding leucine-rich repeat kinase 2, also demonstrated marginally statistically significant associations with depression diagnosis. This finding aligns with previous reports linking *LRRK2* mutations, particularly the G2019S variant, to greater limbic system involvement, a brain region crucial for emotional processing (Belarbi et al. 2010; Chikatimalla et al. 2022). Depression in PD has been associated with denervation of the ventral striatum and mesolimbic dopaminergic pathways, key components of the limbic system responsible for reward processing and emotional regulation (Remy et al. 2005). Although statistical significance was lost after FDR adjustment, consistently low marginal p-values in all analyses support the potential involvement of *LRRK2* in depression among PD patients and a need for further investigation into the gene’s role in mood disorders.

*TRAF5* emerged as another gene associated with depression diagnosis history among European ancestry PD patients. *TRAF5* encodes a tumor necrosis factor receptor-associated factor involved in immune signaling pathways, particularly through the activation of nuclear factor-kappa B (NF-κB) and mitogen-activated protein kinases (MAPKs) (Arch et al. 1998). These pathways are critical in inflammation, cytokine production, and cell survival. Dysregulation of NF-κB signaling has been previously implicated in mood disorders, including depression, likely due to its influence on neuroinflammation and neurotransmitter metabolism (Elhaik and Zandi 2015; Caviedes et al. 2017). Given the increasing evidence linking inflammation to depression in neurodegenerative conditions, the association of SNPs in *TRAF5* with depression in PD suggests a potential role for neuroimmune mechanisms underlying mood disturbances.

### Depression in PD: a distinct genetic mechanism?

Among the depression-related genes previously identified in the general population, SNP sets within *SPPL3* and *ERBB4* showed relatively consistent associations with depression diagnosis and *FNIP2* with current depressive symptoms in our study. *SPPL3*, a signal peptide peptidase-like protein, has been implicated in endoplasmic reticulum (ER) stress (Kuhn et al. 2015) and immune signaling pathways (Li et al. 2018), suggesting potential relevance to neuroinflammation and mood regulation. *ERBB4*, a receptor tyrosine kinase linked to neural development and synaptic plasticity, has previously been associated with psychiatric conditions, including schizophrenia and mood disorders (Chung et al. 2018) and neurodegenerative diseases such as amyotrophic lateral sclerosis (ALS) (Takahashi et al. 2013). Folliculin interacting protein 2 (*FNIP2)* has been identified in GWAS of depression, but its functional relevance to mood disorders remains unclear.

While these genes suggest some overlap between the genetic architecture of depression in the general population and PD, the lack of consistent associations with most previously identified depression-related genes highlights potential differences in the underlying mechanisms. It is possible that depression in PD mainly involves biological pathways, such as neuroinflammation related to neurodegeneration, that are less common in depression in general. This distinction highlights the importance of studying mood disorders in disease-specific contexts, as findings in the general population may not translate to co-morbid forms of depression. Future research, particularly in larger and more diverse cohorts, will be essential to further elucidate these potentially distinct pathways and identify therapeutic targets tailored to the specific context of depression in PD. Additionally, it would be worthwhile to investigate whether genes identified here, particularly *KRTCAP2*, are also associated with other non-motor features of PD such as cognitive impairment or autonomic dysfunction, which could provide broader insights into the role of immune-related pathways across the non-motor spectrum of PD.

Furthermore, our study did not replicate the associations previously reported between depression and PD-related genes, including *RBMS1*,* PCLO*,* ASXL3*,* EP300*,* L3MBTL2*,* CHADL*,* RANGAP1*,* ZC3H7B*, and *TEF* (Reyes-Pérez et al. 2024). While these genes have been suggested as shared genetic factors influencing both PD and major depression, our SKAT analysis yielded large marginal p-values, indicating no statistically significant associations in our dataset. A key reason for this discrepancy is likely the difference in sample size, as the previous study was much larger and may have had greater statistical power to detect associations. In contrast, our study, despite including subjects from multiple studies, remained relatively limited in sample size, particularly for detecting associations driven by rarer variants. Additionally, methodological differences, such as SNP-level vs. gene-based approaches, could have contributed to the variation in results. Further, quality control, particularly LD pruning in our study may have affected our ability to detect haplotype-driven associations.

The depression PRS significantly predicted depression diagnosis in PD patients in both PASIDA and PEG, while the PD PRS did not, suggesting that general depression genetic liability remains relevant in a PD context. Furthermore, our gene-based analysis also identified specific PD risk genes that may contribute to susceptibility of depression in PD patients, particularly immune-related genes such as *KRTCAP2* that did not emerge from the general population depression GWAS. Taken together, depression in PD may therefore reflect a combination of shared depression genetic liability and PD-specific pathways related to neuroinflammation and neurodegeneration.

### Strengths, limitations, and implications

One of the key strengths of this study is our comprehensive approach, leveraging SKAT, Burden tests, and meta-analytical tools across three independent PD cohorts with depression phenotypes. This multi-study design enhances the robustness of our findings, particularly for lower-frequency and rare variants for which single studies are often underpowered.

However, our study is also subject to several limitations. The exclusion of non-coding, singleton, and doubleton variants, and LD pruning may have led to an underestimation of rare variant contributions. Our sample was limited to European ancestry, restricting the generalizability of findings to other populations, and the relatively small sample size compared to large-scale GWAS may have limited our power to detect associations, particularly for rare variants. Participants were not screened for known PD-associated pathogenic variants such as GBA1 and LRRK2 as our goal was to study a general PD population, but the presence of pathogenic variant carriers may introduce some heterogeneity; future studies stratifying by carrier status could provide complementary insights, particularly given the emergence of LRRK2 as relevant for depression in PD in our analyses. Longitudinal analyses were not feasible, as depression diagnosis history was collected at a single time point in PASIDA and PEG. While GDS scores were collected at multiple time points in PEG and PPMI, the number of participants with repeated assessments was insufficient for meaningful longitudinal genetic analyses.

Despite these limitations, our study found gene candidates that, if confirmed, may serve as potential therapeutic targets. The involvement of immune-related pathways, especially those linked to *KRTCAP2*, suggests that anti-inflammatory interventions might be worth exploring as a potential approach for addressing depression in PD patients. While individual genes linked to depression in the general population did not reach statistical significance in our PD cohort - possibly reflecting limited power to detect small individual gene effect sizes - the depression PRS was significantly associated. This underscores that depression in PD shares a polygenic liability with general depression. Similarly, the lack of replication in Hispanic ancestry PD patients suggests that genetic influences may depend on and vary by ancestry.

Further research in larger and more inclusive studies is needed to clarify whether similar immune-related pathways are relevant in other ancestry groups and to advance precision medicine approaches for depression in PD. Additionally, studies with repeated depression assessments over time would allow investigation into whether variants in genes such as *KRTCAP2* are associated with changes in depressive symptoms or other non-motor PD symptoms longitudinally.

## Conclusions

Our gene-based analyses across three PD cohorts identified several PD risk genes that also contribute to depression in PD, highlighting potential roles for immune- and inflammation-related pathways. Furthermore, our depression PRS findings indicate that depression in PD shares a polygenic genetic liability with major depression. While larger and more diverse studies are needed, our work provides a foundation for future investigations into biologically informed approaches for identifying and treating depression in PD.

## Supplementary Information

Below is the link to the electronic supplementary material.

Supplementary Material 1

## Data Availability

The datasets generated and/or analyzed from the PASIDA and PEG studies are available from the corresponding author on reasonable request. Data used from the PPMI study in the preparation of this article were obtained on [2023-09-20] from the Parkinson’s Progression Markers Initiative (PPMI) database (https://www.ppmi-info.org/access-data-specimens/download-data), RRID: SCR_006431. For up-to-date information on the study, visit http://www.ppmi-info.org.
